# Boron-Doped Pine-Cone Carbon With 3D Interconnected Porosity for Use as an Anode for Potassium-Ion Batteries With Long Life Cycle

**DOI:** 10.3389/fchem.2022.953782

**Published:** 2022-07-06

**Authors:** Jian-Fang Lu, Ke-Chun Li, Xiao-Yan Lv, Hong-Xiang Kuai, Jing Su, Yan-Xuan Wen

**Affiliations:** ^1^ School of Chemistry and Chemical Engineering, Guangxi University, Nanning, China; ^2^ School of Chemistry and Chemical Engineering, Guangxi Minzu University, Nanning, China; ^3^ School of Materials and Environment, Guangxi Minzu University, Nanning, China; ^4^ The New Rural Development Research Institute, Guangxi University, Nanning, China; ^5^ Guangxi Key Laboratory of Processing for Non-ferrous Metallic and Featured Materials, Guangxi University, Nanning, China

**Keywords:** potassium-ion batteries, anodes, biomass carbon, pine-cone, boron doping, molten ZnCl_2_

## Abstract

Potassium-ion batteries (KIBs) have received widespread attention as an alternative to lithium-ion batteries because of their low cost and abundance of potassium. However, the poor kinetic performance and severe volume changes during charging/discharging due to the large radius of potassium leading to low capacity and rapid decay. Therefore, development of anode materials with sufficient space and active sites for potassium ion deintercalation and desorption is necessary to ensure structural stability and good electrochemical activity. This study prepared boron-doped pine-cone carbon (BZPC) with 3D interconnected hierarchical porous in ZnCl_2_ molten-salt by calcination under high temperature. The hierarchical porous structure promoted the penetration of the electrolyte, improved charge-carrier diffusion, alleviated volume changes during cycling, and increased the number of micropores available for adsorbing potassium ions. In addition, due to B doping, the BZPC material possessed abundant defects and active centers, and a wide interlayer distance, which enhanced the adsorption of K ions and promoted their intercalation and diffusion. When used as the anode of a KIB, BZPC provided a high reversible capacity (223.8 mAh g^−1^ at 50 mA g^−1^), excellent rate performance, and cycling stability (115.9 mAh g^−1^ after 2000 cycles at 1 A g^−1^).

## Introduction

With increasing global demand for clean energy, the development of renewable and non-polluting energy systems has become a key driver of human development. However, the intermittent and variable output of renewable-energy sources can adversely impact grid stability and thus hinder their widespread use (Holdren, 2007; Yang et al., 2011). Energy storage systems (ESSs) are being extensively developed to improve grid stability in the form of mechanical, electrical, electrochemical, thermal, and chemical energy storage (Holdren, 2007; Yang et al., 2011). Lithium-ion batteries (LIBs) are among the most notable ESSs due to their high energy density and long cycle life (Guo et al., 2021; Hyun et al., 2021; Li et al., 2021). However, the large-scale application of LIBs in electric, hybrid vehicles and smart grids has put pressure on the limited supply of lithium resources. LIBs also have other limitations, such as high cost and potential safety hazards (Wang et al., 2018a; Wu et al., 2020). Many researchers attempted to develop alternative ESS with high energy and power density to address these issues. Potassium-ion batteries (KIBs) are receiving increasing attention because potassium is abundant, exhibiting similar physicochemical properties to lithium, and the potassium-ion batteries possess superior safety performance (Hosaka et al., 2020; Rajagopalan et al., 2020; Yao and Zhu, 2020). However, because potassium ions have a much larger radius (1.38 Å) than lithium ions (0.76 Å), they have a higher resistance to the insertion/extraction processes in the anode material, resulting in irreversible electrode material distortion, as evidenced by low potassium storage capacity, low Coulombic efficiency in the first cycle, and rapid capacity decay during long-term cycling (Lian et al., 2022). Therefore, optimization of anode materials is essential for developing potassium-ion batteries.

To date, various materials have been developed and tested as anode materials for KIBs (Rajagopalan et al., 2020), such as carbon materials (Wang et al., 2018b; Zhang et al., 2019; Yang et al., 2020; Zhao et al., 2020), alloys (Zhang et al., 2018), metal oxides (Liu et al., 2021), and metal sulphides (Hu et al., 2021). Among these materials, carbon-based materials are ideal candidates for the anode of KIBs, because of their low price, adequate electrical conductivity, and stable structure (Jian et al., 2015; Zhang et al., 2019; Rajagopalan et al., 2020). Carbon materials with different structures and morphologies have been extensively reported in the literature for KIB anodes, including hard carbon (Yamamoto et al., 2018; Lin et al., 2020), graphene (Tung et al., 2022), carbon nanotubes (Liu et al., 2018), and carbon nanospheres (Lu et al., 2019). However, most of them have poor cycling stability, rate performance, and limited specific capacity, which mainly due to extensive volume changes from intercalation of the large potassium ions. Various strategies have been developed to improve the electrochemical performance of carbon materials. For instance, 3D-interconnected hierarchical porous structures providehigher accessible surface area to facilitate adequate contact between the electrode material and electrolyte (Li et al., 2017; Tao et al., 2020). This shortens the diffusion channels for ions and electrons and alleviates the volume changes in the material caused by the insertion/extraction of ions with large radii (Wang et al., 2018a; Li et al., 2019; Cui et al., 2020a), improving the storage of potassium ions. In addition, the introduction of heteroatoms into the carbon skeleton is an effective strategy for improving its electrochemical properties (Wang et al., 2018a; Liu et al., 2018; Li et al., 2019; Cui et al., 2020a; Cui et al., 2020b; Liu et al., 2020). Heteroatom doping of the carbon lattice induces more defects and active sites, widens the interlayer spacing, and increases the electrical conductivity to improve potassium-storage performance [6 28]. Among the reported atoms used for doping, boron atoms have a similar radius to carbon atoms and can be easily introduced into the carbon skeleton. Additionally, as an electron-deficient atom, boron can activate inert electrons in the carbon skeleton after being introduced into the carbon skeleton, leading to sufficient charge transfer from potassium to the substrate. Therefore, boron is an ideal dopant and has received extensive attention. For instance, three-dimensional (3D) boron-doped carbon structures (Wang et al., 2018a; Cui et al., 2020a), boron-doped graphene (Wang et al., 2016), and boron-doped layered aggregated carbon particles (Lian et al., 2022) have been used as anode materials for LIBs, KIBs, and sodium-ion batteries, providing high capacity and good cycling stability. To the best of our knowledge, Few literatures reported about boron-doped carbon materials as anode electrodes for potassium ion batteries.

Highly abundant Biomass is widely distributed worldwide. Biomass such as agricultural waste is traditionally disposed by combustion, resulting in the waste of useful resource and severe environmental pollution. Therefore, it still remains of essential significance to explore a new approach to improve the utilization value of bagasse and meet the requirement of green economy. For example, biomass has been used as a carbon source to prepare various valuable carbon materials for electrochemical energy storage applications (Cui et al., 2020a; Lin et al., 2020; Tao et al., 2020; Yang et al., 2020).

Based on the above-mentioned background, porous carbon materials from pine-cone biomass were fabricated as anode materials of KIBs. 3D interconnected hierarchical porous carbon was prepared by one-step calcination in the presence of molten zinc chloride salt and boric acid, providing boron-doped carbon. This preparation process was chosen as it is simple, easy to control, and uses basic low-cost equipment and is expected to provide a feasible and controllable method for preparing doped carbon materials.

## Materials and Methods

### Material Preparation

First, 3 g of pine-cone powder (taken from the Shishan Park, Nanning, China), 12 g of ZnCl_2_, and 3 g of boric acid were added to 50 ml of distilled water and stirred magnetically for 12 h. After the solvent was evaporated, the materials were dried, ground, and mixed well. Then, the mixture was heated to 800 °C under a nitrogen atmosphere at a rate of 5°C min^−1^, held for 5 h, and cooled to room temperature. The thus-obtained material was soaked in 30% hydrochloric acid for 3 h, and then washed repeatedly with 80°C distilled water to remove the excess zinc chloride and other residual impurities. The remaining carbon was boron-doped pine-cone carbon (called BZPC). Various control materials were prepared for comparison: pine-cone carbon prepaeed (ZPC) and boron-doped pine-cone carbon (BPC) were prepared in the same way as the BZPC, except with the addition of only ZnCl_2_ or boric acid, respectively. In addition, pure carbon (PC) was prepared without any dopant.

### Material Characterisation

A D8 Advance X-ray diffractometer (Bruker, Germany) was used for phase analysis. Field-emission scanning electron microscopy (FESEM; SUPRA 55Sapphire, Zeiss, Germany) and transmission electron microscopy (TEM; FEI Tecnai G2 F20s twin 200 kV, United States ) and high-resolution transmission electron microscopy (HRTEM; FEI Tecnai G2 F20s-twin 200 kV, United States ) were used to observe the morphology and microstructure of the materials. The specific surface area and pore size distribution of the materials were analysed using a Micromeritics TriStar II 3020 specific surface and porosity analyser (Micromeritics Instruments Corporation, Georgia, United States ). The specific surface area was calculated using the BET method. The microporous specific surface area (S_mic_) and micropore volume (V_mic_) were calculated by the t-plot method. Pore volume, and pore size distribution of the materials were determined by the NLDFT and BJH models. An inVia confocal Raman spectrometer (Renishaw, United Kingdom), was used to analyse the degree of graphitisation of the materials. The test laser wavelength was 532 nm and the degree of graphitisation was determined from the ratio of the intensities of the D and G peaks (I_D_/I_G_) in the measured Raman spectra. X-ray photoelectron spectrometry (XPS; ESCALAB 250XL+) was used to qualitatively and quantitatively analyse the species and valence states of the elements in the carbon materials.

### Electrochemical Tests

The carbon material, acetylene black, and polyvinylidene fluoride (PVDF) binder were ground in an agate mortar in a mass ratio of 8:1:1, respectively. The mixture was mixed with a certain amount of N-methylpyrrolidone (NMP) and an appropriate amount of agate balls and then ball milled in a high-speed oscillating ball mill for 8 min. The mixed slurry was coated on copper foil, dried under vacuum at 120 °C for 12 h, and then stamped into electrode samples (14 mm diameter). The amount of active substance is about 1.5–2.0 mg cm^−2^.

The prepared electrodes were assembled into a 3020-type button cell battery in a glove box in a high-purity argon atmosphere. In electrochemical tests, the as-prepared carbon electrodes were used as the working electrodes, potassium metal electrodes were used as counter electrodes, glass fibre (Whatman, GF/B) was used as a diaphragm, and the electrolyte was 0.8 M KPF_6_/ethylene carbonate (EC)/diethyl carbonate (DEC) (1:1 volume ratio). The cells were tested after they were left to sit at room temperature for 8 h.

Constant-current charge and discharge tests were performed using a BTS 4000 system (Neware Electronics Co., Ltd., Shenzhen, China) at a temperature of 25 °C over a voltage range of 0.01–3 V (vs K/K^+^). The current density and specific capacity of the samples were calculated based on the carbon mass of the working electrode. A PCI 4750 electrochemical workstation (Gamry, United States) was used to perform cyclic voltammetry (test temperature: 25°C, scan rate: 0.1, 0.2, 0.5, 0.7, 1, 1.5, and 2 mV s^−1^; voltage range: 0.01–3 V) and electrochemical impedance spectroscopy (EIS; temperature: 25°C; frequency: 10 mHz to 100 kHz; amplitude of AC voltage: 5 mV).

## Results and Discussion

### Structural and Morphological Characterization

**FIGURE 1:**
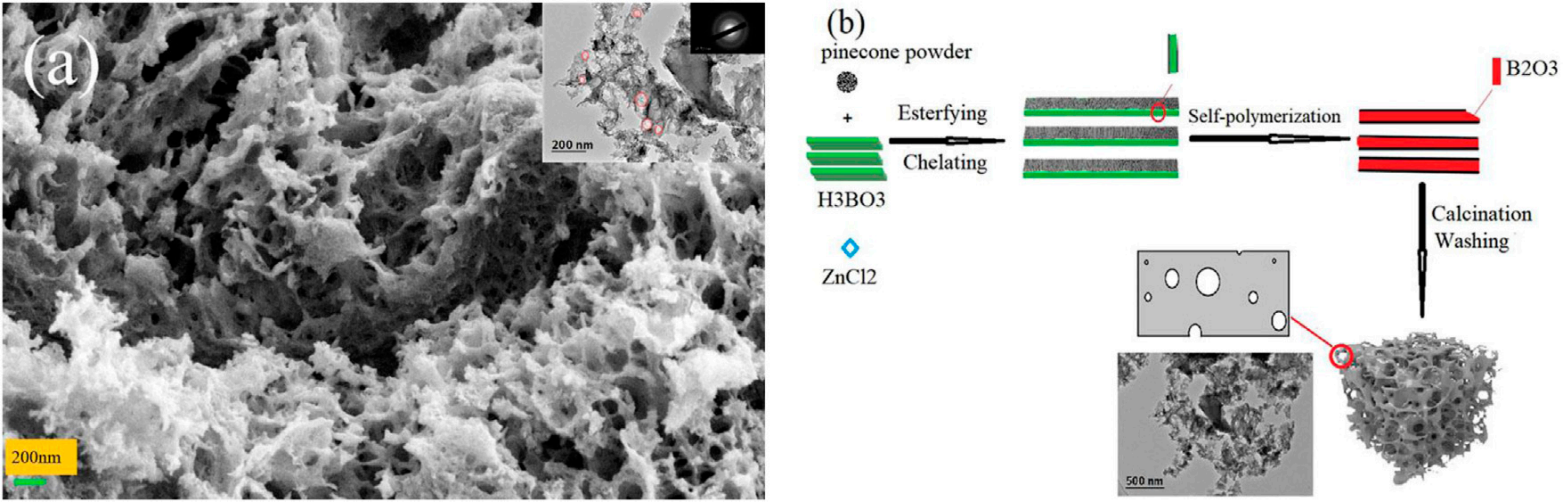
**(A)** SEM and (Inset) TEM images of BZPC, **(B)** Schematic illustration for the formation mechanism of a thin BZPC sample.

Four types of samples were prepared to investigate the effect of boric and zinc doping on the microstructure of pine-cone carbon. Figure 1A and Supplementary Figures S1A–C showed the SEM and TEM (inset) images of the four samples. ZPC (Supplementary Figure S1A) comprised large blocks composed of fine particles; BZPC (Figure 1A) showed a 3D interconnected porous structure; BPC (Supplementary Figure S1B) had blocky structure with a rough surface and were comprised of short rod-like particles; and PC (Supplementary Figure S1C) had an uneven surface with irregular cavities. The TEM images showed multiple overlapping carbon layers in ZPC, BPC, and PC, while BZPC contained ultra-thin carbon layers with pores of different sizes; the above latter should facilitate electrolyte penetration and rapid electron and ion transport (Li et al., 2017; Wang et al., 2018a). The SEM/TEM results confirmed that the porous structure and ultra-thin carbon layers of BZPC were formed by boron doped and molten ZnCl_2_. Based on these results, the formation mechanism of the ultrathin carbon layer of BZPC has been proposed (Figure 1B). After the pine-cone powder was impregnated with boric acid solution, boric acid molecules penetrated the cell walls and cavities of the lignocellulosic biomass or were deposited on the surface of the biomass powder, forming non-covalently bonded complexes with hydroxyl groups of lignocellulose through hydrogen bonding (Zhang et al., 2020a; Hou et al., 2021; Le et al., 2021). The boric acid underwent self-polymerisation at the start of pyrolysis and eventually formed a glassy B_2_O_3_ film on the lignocellulose surface (Hou et al., 2021; Le et al., 2021). Then, lignocellulose went through a series of pyrolysis reactions during calcination and eventually forming a thin localised carbon layer. The formation of the specific pore structure was due to the combined effect of boric acid and zinc chloride, which will be described in detail later.

**FIGURE 2:**
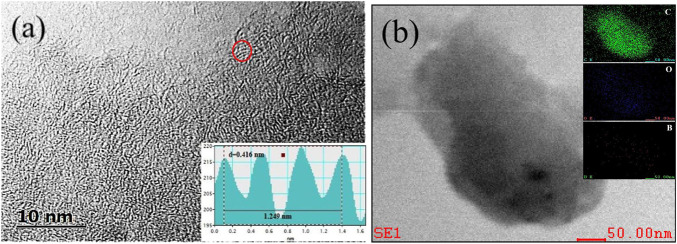
**(A)** HRTEM image and **(B)** EDS map of BZPC.

High-resolution TEM (HRTEM) image (Figure 2A) also indicated a highly disordered structure with a small amount of local weakly ordered framework in BZPC, suggesting an amorphous structure. This was consistent with the results of selected area electron diffraction (SAED, inset of Figure 2A). Compared with pure carbon (ZPC) ((Supplementary Figure S2A), doped boron broadened the carbon framework, expanded the average distance between graphite layers from 0.342 nm (ZPC) to 0.416 nm (BZPC) (Wang et al., 2016; Ahmad et al., 2020), which was conducive to potassium ion insertion/extraction. Furthermore, compared with the energy dispersive X-ray spectroscopy (EDS) map of ZPC, the boron signal of BZPC was uniformly distributed throughout the carbon matrix, indicating that boron was successfully doped into the carbon skeleton (Figure 2B and Supplementary Figure S2B).

Supplementary Figure S3A displayed the XRD spectra of BZPC and ZPC, both materials showed amorphous carbon features with two broad characteristic diffraction peaks around 23° and 42° corresponding to the 002 and 100 planes of graphitic carbon (Gao et al., 2019; Ahmad et al., 2020), respectively. These results were consistent with the HRTEM images. The (002) peak of BZPC shifted towards lower 2θ relative to that of ZPC, indicating expanded layer spacing in BZPC (Liu et al., 2018; Lian et al., 2022). Based on the Bragg equation, the interlayer distances of BZPC and ZPC were calculated as 0.418 and 0.345 nm, respectively, which were in general agreement with the HRTEM data. This revealed that the B-doped carbon framework had an enlarged interlayer spacing, which should facilitate K^+^ insertion/extraction (Wang et al., 2016). It was speculated that B was doped into the carbon skeleton at defective sites or edges to form either BCO_2_ or BC_2_O with strong B–O bonds acting as a backbone for the expanded interlayer spacing (Ni et al., 2019).

Supplementary Figure S3B showed Raman spectra of BZPC and ZPC, where the two strong and broad overlapping peaks near 1,356 and 1,587 cm^−1^ correspond to the D-band and G-band, respectively (Lian et al., 2022). The D-band is associated with the in-plane carbon atom vibrations in the dangling band of disordered graphite, reflecting the characteristics of a disordered or defective structure. The G-band is related to the atomic stretching vibrations of sp^2^ bonded carbon atoms in the hexagonal lattice, reflecting the characteristics of a crystalline or ordered graphite structures. Thus, the ratio I_D_/I_G_ is used to indicate the degree of disorder in carbon materials (Yang et al., 2020). The I_D_/I_G_ values of BZPC and ZPC were 1.03 and 0.89, respectively, indicating that B doping increased the disorder in the carbon material, exposing more defect sites and providing more active sites for the adsorption and desorption of potassium ions, therefore improving capacitive storage and rate capability (Wang et al., 2018a; Cui et al., 2020a). The introduction of boron primarily created more edges, defects, and vacancies in the carbon skeleton, which inhibits graphitization, resulting in an increase in carbon disorder (Wang et al., 2019; Ahmad et al., 2020).

**FIGURE 3:**
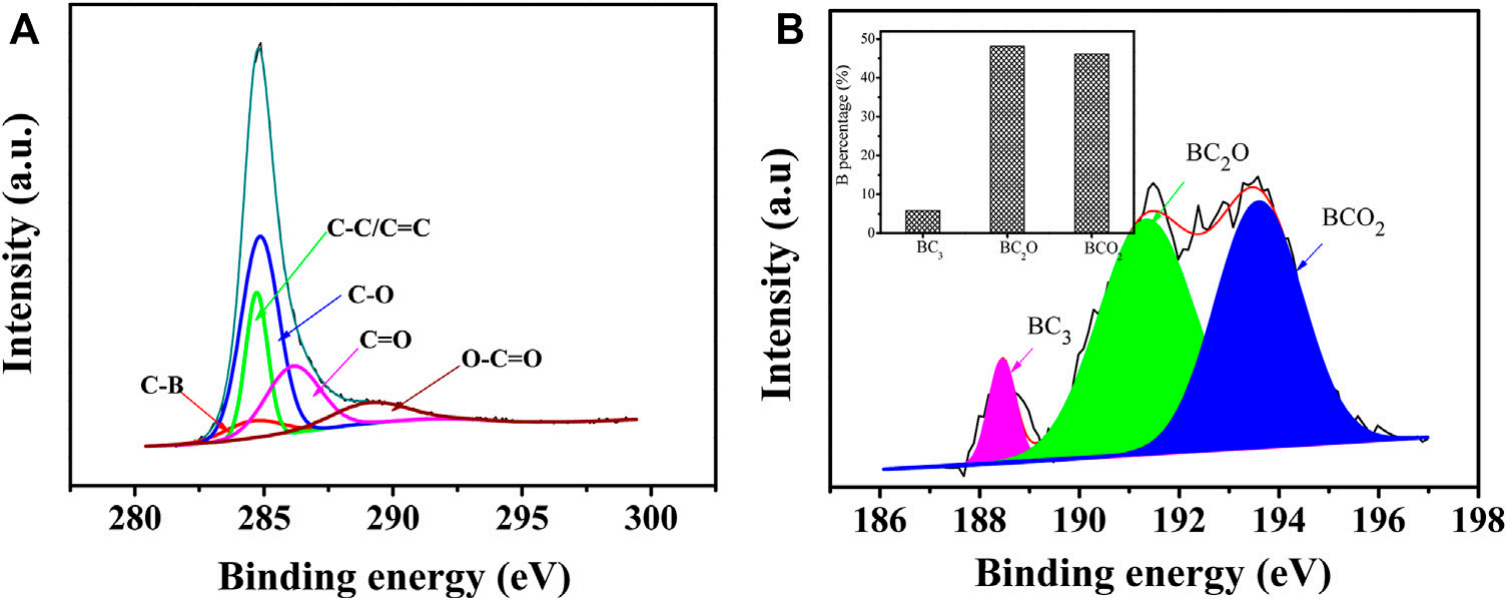
**(A)** High-resolution C1s spectra of BZPC. **(B)** High-resolution B1s spectra of BZPC (Inset: relative ratios of BC_3_, BC_2_O, and BCO_2_).

**FIGURE 4:**
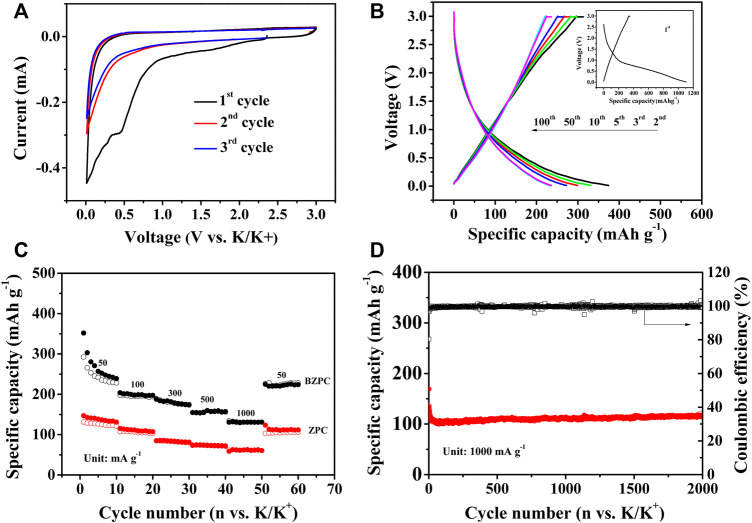
**(A)** CV curves of BZPC at 0.1 mV s^−1^, **(B)** Galvanostatic charge/discharge profiles of different cycles of BZPC at 50 mA g^−1^ (Inset: 1st cycle), **(C)** Rate performance and **(D)** Long-term cycle stability of BZPC.

XPS can be used to further understand the surface chemical composition of the material and the chemical bonding. Supplementary Figure S4A showed the full XPS spectra of BZPC and ZPC, where the C1s and O1s peaks were observed with binding energies of approximately 285 and 531 eV, respectively. In contrast to ZPC, the spectrum of BZPC had a tiny peak at a binding energy of ∼191 eV, which is a B1s peak with 1.63% atomic content, indicating successful boron doping (Wang et al., 2018a). Compared with the high-resolution C1s deconvolution peak of ZPC (Supplementary Figure S4B), BZPC has an additional fitted peak at 284.1 eV corresponding to the C–B bond (Figure 3A) (Ahmad et al., 2020), further confirming the binding of B atoms to the carbon skeleton. In addition, the high-resolution B1s peak of BZPC was deconvoluted into three characteristic peaks (Figure 3B) at 188.5, 191.35, and 193.6 eV, corresponding to BC_3_, BC_2_O, and BCO_2_, respectively (Wang et al., 2016; Wang et al., 2019; Lian et al., 2022). In the BC_3_ structure, B replaced a carbon atom in the six-membered ring. As B atoms are electron deficient, they can generate positively charged holes to transfer electrons from the carbon structure, thus improving the electrical conductivity of the material (Wang et al., 2016; Wang et al., 2019; Ahmad et al., 2020). In the BC_2_O and BCO_2_ structures, the B atom replaces the C atom at the edge or defect of the carbon structure. These 2 B groups widen the lattice spacing and introduced many defects and active sites, all of which facilitate the insertion, adsorption, and rapid transfer of K ions (Wang et al., 2016; Wang et al., 2019; Ahmad et al., 2020). The inset in Figure 4B showed the B percentage of the three structures. The content of B in BC_2_O and BCO_2_ was significantly higher than that in BC_3_, indicating that B atoms are mainly located at the edges/defects in the carbon structure. It is also noteworthy that a significant increase in oxygen content in BZPC compared to ZPC was observed, suggesting enhanced oxygen grafting by boron doping (Sun et al., 2018). When B is doped into a carbon planar structure, it causes the redistribution of π electrons in the host structure, weakening the C–C bonds and strengthening the C–O bond, due to its low electronegativity (2.04) relative to that of C (2.55) (Wang et al., 2008; Sun et al., 2018). Therefore, higher oxygen concentrations are expected in boron-doped materials. The oxygen-containing groups also provide active sites for the storage of K ions (Wang et al., 2018a; Zhang et al., 2021; Lian et al., 2022).

The isothermal adsorption-desorption curves for BZPC and ZPC and their corresponding pore-size distributions were shown in Supplementary Figure S5. The specific surface area and pore structure parameters extracted from these data were presented in Table 1. Supplementary Figures S5A,C showed that the adsorption-desorption isotherms for both BZPC and ZPC are type-IV isotherms (Li et al., 2020). A clear hysteresis loop within a relative pressure range of 0.4 and 1.0 indicated the presence of a mesoporous structure (Wang et al., 2018a; Zhang et al., 2020b). When the relative pressure is 0–0.05, the adsorption curve rose sharply, implying the presence of micropores [27]. In addition, the BZPC curve showed a slow increase between 0.45 and 0.99, suggesting a small number of macropores (Wang et al., 2008). The micropores of BZPC were mainly around 0.35 and 0.5 nm, the mesopores were in the range of 2–6 nm and 20–50 nm, and there were few large pores. The micropores of ZPC were around 0.5 nm, while mesopores were mostly 2–12 nm. The pore-size distributions (Supplementary Figures S5B,D) also showed that the micropores of BZPC had a stronger adsorption effect compared to ZPC, indicating higher microporosity. These results indicated that ZPC was a hierarchical porous structure consisting of micropores and mesopores, while BZPC was a hierarchical porous structure composed of micropores, mesopores, and macropores. The micropores ensure a larger contact area between the electrolyte and active material; the mesopores promote rapid diffusion of electrons and ions; and the macropores act as reservoirs to facilitate the storage of many potassium ions at high rates (Yu et al., 2015). Table 1 confirmed that the BET surface area increased upon boron doping, from 1,697 m^2^ g^−1^ (ZPC) to 1976 m^2^ g^−1^ (BZPC), which increases the electrolyte-electrode interfacial area. The micropore area increased from 393 m^2^ g^−1^ (ZPC) to 963 m^2^ g^−1^ (BZPC) and the micropore content almost doubled (25.32% for BZPC), conducive to the adsorption of K ions (Lian et al., 2022). In addition, BZPC contained few macropores. Therefore, the hierarchical porous structure of BZPC is expected to be more effective than the other carbon materials for improving the electrochemical performance of pine-cone carbon, especially the rate capability.

**TABLE 1:** Basic physical-chemical properties of ZPC and BZPC.

Sample	*S*_BET_ (m^2^g^−1^)	S_micro_ (m^2^g^−1^)	S_meso_ (m^2^g^−1^)	S_macro_ (m^2^g^−1^)	*V*_tot_ (cm^3^g^−1^)	*V*_micro_ (cm^3^g^−1^)	*V*_meso_ (cm^3^g^−1^)	*V*_macro_ (cm^3^g^−1^)	*V*_micro_ */V*_tot_ (%)	Content (at%) from XPS			I_D_/I_G_
										C	O	B	
ZPC	1,697	393	1,304	0	1.66	0.24	1.42	0	14.46	94.39	5.60	0	0.89
BZPC	1976	963	815	198	1.78	0.45	1.23	0.1	25.32	91.46	6.92	1.63	1.03

Boric acid plays multiple roles in the formation of the pore structure. First, B–OH reacts with hydroxyl groups to produce water (Zhang et al., 2020a). Second, during carbonation, boric acid catalyses the dehydration and deoxygenation of lignocellulose, releasing water, CO, CO_2_, and some volatile small molecules (Zhang et al., 2020a; Hou et al., 2021; Le et al., 2021). These lead to the formation of a microporous structure. Finally, boric acid also acts as a template. As the temperature increased during calcination, boric acid is gradually dehydrogenated and deoxidized, eventually producing B_2_O_3_ and water. Micropores were formed during the subsequent evaporation of the water. B_2_O_3_ has been used as a template to form large mesopores and macropores (Cui et al., 2020a; Jin et al., 2020; Li et al., 2020; Wang et al., 2020). Zinc chloride also played two roles in the formation of the porous structures. It acted as a dehydrating agent to remove hydrogen and oxygen atoms from the pine-cone powder through cycloaddition and degradation reactions. The hydrogen and oxygen atoms escaped as water molecules and formed micropores (Donald et al., 2011; Yahya et al., 2015).

### Energy-Storage Performance and Mechanism

Supplementary Figure S6A and Figure 4A showed CV curves of ZPC and BZPC at a scan rate of 0.1 mV s^−1^. The CV curves of the samples were similar except for the first cycle, indicating that both carbon samples had a similar K-ion storage mechanism. The sharp cathodic peak at ∼0.01 V was typical of the insertion of K ions into the graphite layer to form KC_x_ compounds (Gao et al., 2019; Lian et al., 2022). The corresponding anodic peak was broad (over a wide voltage window), which is attributed to the adsorption behaviour of abundant large-radius potassium ions at the active/defect sites of the electrode material and the slow electrochemical reaction kinetics (Ruan et al., 2019; Deng et al., 2021). Furthermore, an irreversible reduction peak near 0.5 V was observed for ZPC and BZPC in the first cycle of the CV curve, which was not observed in subsequent scans, corresponding to the decomposition of the electrolyte on the electrode surface to form a solid electrolyte interphase (SEI) film and the irreversible insertion of K ions (Ruan et al., 2019; Deng et al., 2021; Lian et al., 2022). Additionally, compared to ZPC, the weak irreversible peak of BZPC indicated that B doping increased the Coulombic efficiency.

Supplementary Figure S6B and Figure 4B showed the charge and discharge curves for ZPC and BZPC at the 1st, 2nd, 3rd, 5th, 10th, 50th, and 100th cycles (see inset for the first cycle). The initial discharge curve showed a voltage plateau near 0.5 V, corresponding to the irreversible reduction peak of the first cycle in Supplementary Figure S6A and Figure 4A (Ruan et al., 2019; Deng et al., 2021), which was not observed in subsequent cycles. The first-cycle discharge/charge specific capacities of ZPC and BZPC were 691.9/94.7 and 1,105.4/351.2 mAh g^−1^, respectively, with corresponding initial Coulombic efficiencies of 13.68 and 31.76%. Compared to ZPC, the increase in initial Coulombic efficiency of BZPC was due to the introduction of B into the carbon skeleton, adding more active sites and widening the interlayer spacing, which reduced the irreversible insertion/extraction of potassium ions (Wang et al., 2018a; Lian et al., 2022).

Supplementary Figure S6C and Figure 4C displayed the cycling performance and rate capability, respectively, for the BZPC and ZPC electrodes. Supplementary Figure S6C indicated that after 100 cycles at 50 mA g^−1^, BZPC can provide 235.9 mAh g^−1^ discharge specific capacity, while ZPC only provides 94.4 mAh g^−1^. BZPC showed high specific capacity and superior cycling performance, primarily due to its large specific surface area and pore volume, which facilitates the electrochemical reactions and diffusion/penetration of the electrolyte (Wang et al., 2018a; Liu et al., 2018; Deng et al., 2021). The high content of BC_2_O and BCO_2_ in BZPC could generate more active and defect sites (Wang et al., 2008; Wang et al., 2019; Ahmad et al., 2020) to provide additional reversible storage sites for K ions. In addition, the formation of BC_2_O and BCO_2_ increases the interlayer spacing (Wang et al., 2016; Ahmad et al., 2020), which is conducive to the insertion/extraction of potassium ions (Wang et al., 2018a). As shown in Figure 4C, the discharge specific capacities of BZPC electrodes were 238.7, 197.2, 174.4, 156.8 and 130.9 mAh g^−1^ at current densities of 50, 100, 300, 500 and 1,000 mA g^−1^, respectively. These values were approximately 2.5 times those of ZPC under the same conditions, indicating the effective increase in the rate capability of pine-cone carbon by B doping. In addition, ZPC could last 530 cycles with the discharge specific capacity of 70.4 mAh g^−1^ at 1 A g^−1^, and then the capacity decayed significantly (Supplementary Figure S6D), while the discharge specific capacity of BZPC remained at 115.9 mAh g^−1^ after 2,000 consecutive cycles, with a corresponding capacity retention of 68.5% and a capacity decay as low as 0.03% cycles, showing adequate long-term cycling stability (Figure 4D). The long cycle stability of BZPC has certain advantages compared with the reported B-doped carbon. For example, Lian et al. (Lian et al., 2022) prepared Boron/Oxygen co-doped carbon particles via plasma-enhanced chemical vapor deposition using hydroxyapatite as growth template and trimethyl borate as the precursor. The carbon particles delivered a capacity of 120.0 mAh g^−1^ after 500 cycles at 1A g^−1^. Liu et al. (Liu et al., 2022) synthesized B, N co-doped porous hollow multi-cavity carbon spheres. The carbon showed 75.3% capacity retention from cycle 11 to 2000 at 1 A g^−1^, while BZPC dispalyed no capacity loss in the same state. The improved performance for BZPC was mainly attributed to the ultra-thin carbon layer, which favoured electrolyte penetration and promoted rapid electron and ion transport (Li et al., 2017; Wang et al., 2018a), and the 3D interconnected pore structure, which enabled sufficient contact between the electrolyte and active material, shorten the diffusion path of electrons and ions and alleviated the volume expansion of the electrode materials due to the insertion/extraction of large potassium ions (Li et al., 2017; Ahmad et al., 2020).

**FIGURE 5:**
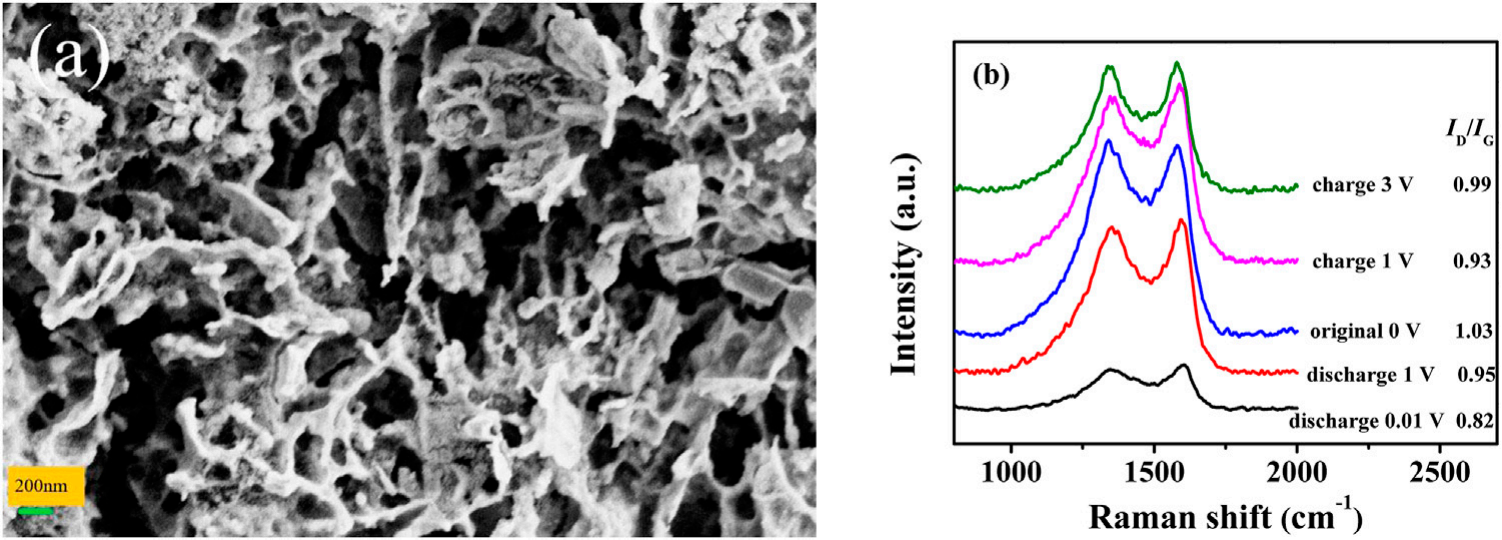
Effect of cycling on the anode microstructure. SEM images of **(A)** BZPC after 1,000 cycles before testing. **(B)** *Ex-situ* Raman spectra of BZPC at various states.

To understand the effect of B doping, *ex-situ* SEM/HRTEM was used to characterise the morphological changes of ZPC and BZPC in different charge/discharge states. Figure 5A and Supplementary Figure S7A showed SEM images of BZPC and ZPC after 1,000 charging and discharging cycles. Compared with Figure 1A and Supplementary Figure S1a, the primary particle agglomeration of ZPC became larger after 1,000 cycles, which is not conducive to embedding potassium ions. In contrast, BZPC mostly maintained its original favourable structure, indicating structural stability during cycling. Supplementary Figures S7B–D displayed the HRTEM images of the initial material, as well as the fully charged and discharged states after 10 cycles, respectively. The original average interlayer spacing of BZPC was 0.413 nm (Supplementary Figure S7B), which increased significantly to 0.469 nm after complete discharge (Supplementary Figure S7C), indicating the lattice expansion caused by the successful insertion of K ions into BZPC. When K ions were extracted from the carbon structure, the average interlayer spacing decreased again to 0.426 nm (Supplementary Figure S7D). Subsequently, the interlayer spacing only changed 3.1% during charge/discharge cycling, revealing the excellent reversibility and structural stability of the electrode material during insertion/extraction of K ions. Notably, when K ions were embedded in the carbon skeleton, locally disordered regions were transformed into ordered graphite stacking layers. Comparing Supplementary Figures S7E,F, the dramatic change in K content between the fully charged and fully discharged states further indicated that the potassium ions were successfully inserted and extracted from the carbon matrix. However, the K content did not fall to zero after complete charging, implying an incomplete detachment of K ions from the BZPC electrode, possibly due to residual potassium as an SEI layer and irreversible deintercalation or desorption of K ions in the active centres (Cui et al., 2020b). *Ex situ* XPS was further applied to investigate the changing level of K inside the BZPC electrode during the intercalation/deintercalation process and the fresh state. As shown in Supplementary Figure S7G, no K-signal peaks were observed in the fresh BZPC electrode. However, two characteristic peaks (K 2p3/2 and K 2p1/2) were detected in full discharge state, indicating that potassium ions were intercalated into BZPC. In the subsequent full charge state, the K 2p peak intensity decreased as potassium ions were extracted reversibly from the carbon framework. These results were consistent with the EDS-mapping observations in Supplementary Figure S7E,F. Thus, the good performance of BZPC can be attributed to the reversible potassiation/depotassiation processes and the stable microstructure.

In addition, the structural evolution of BZPC after 10 cycles was analysed using *ex-situ* Raman spectroscopy (Figure 5B). The I_D_/I_G_ ratio gradually decreased from 1.03 to 0.88 as the discharge voltage dropped from the initial state to 0.01 V, revealing the transformation of the localised disordered carbon region into a graphitic stacking region due to K ion insertion (Wang et al., 2018b; Wu et al., 2019a). This is consistent with the *ex-situ* HRTEM observations. When charging was complete, I_D_/I_G_ returned to 0.99 (initial value was 1.03), indicating that the potassium ion deintercalation increased the graphitization of the carbon matrix and potassium ions remained in the graphite layer (Cui et al., 2020b; Zhang et al., 2020b). These results indicated that the excellent electrochemical properties of BZPC might be attributable to the reversible insertion/extraction of potassium ions and the stable microstructure.

**FIGURE 6:**
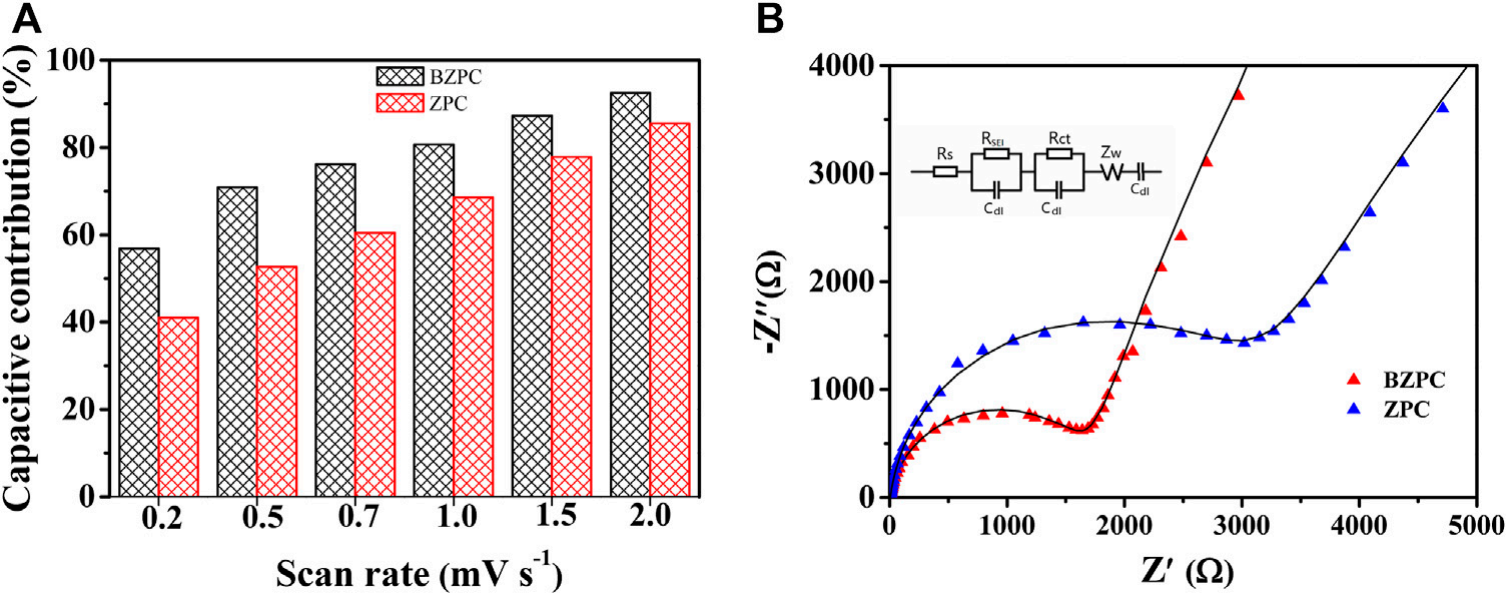
**(A)** Capacitive contributions at various scan rates from 0.2 to 2 mV s^−1^, and **(B)** Nyquist plots of ZPC and BZPC (Inset: equivalent circuit diagram).

The previous results showed that the capacity of ZPC and BZPC was due to both the intercalation of potassium ions in the carbon layer and their adsorption at active sites (He et al., 2020). Therefore, CV at different scan rates was used to analyse the effect of B doping on the mechanism of charge storage. Supplementary Figure S8a,b showed CV curves for BZPC (0.2–2 mV s^−1^) and ZPC (0.01–3 V), respectively. The CV curves for ZPC and BZPC between 1.25 and 3 V were rectangular, indicating the capacitive behaviour of the potassium-ion storage process (Lian et al., 2022), in agreement with the results of the charge–discharge curves. It is well known that the relationship between current and potential scan rate in CV can be expressed by log(*i*) = *b*log(*v*)+log(*a*), where *i* is the peak current, *v* is the scan rate, and *a* and *b* are fitting constants (Wang et al., 2018a; Lian et al., 2022). Based on this relationship, the *b* values of the cathodic peaks were calculated as 0.9014 and 0.9445 for ZPC and BZPC, respectively; these values are between 0.5 and 1, indicating that potassium storage in these materials involves both diffusion-controlled and surface-controlled processes (Li et al., 2017; Liu et al., 2020). Additionally, the capacitive contribution was quantified using *i*(*V*) = *k*_1_ *ν + k*_2_ *ν* ^1/2^, where *k*_1_ *v* and *k*_2_ *v* ^1/2^ are the capacitive and diffusive contributions, respectively (Liu et al., 2020). Figure 6A compared the capacitive contribution of BZPC and ZPC. The capacitive contribution of BZPC and ZPC increased with the increase of the potential scanning rate. When the scan rate increased from 0.2 to 2

mV s^−1^, the capacitive contribution of BZPC increased from 56.8 to 92.5%. The capacitive contribution of BZPC was 10–20% higher than that of ZPC for the same scan rate. Thus, these results further confirmed that the storage behaviour of K ions depended strongly on surface-controlled (adsorption) processes. The high capacitive contribution of BZPC may be attributed to its large specific surface area and the abundant surface defects and active sites at the carbon edges (Wang et al., 2018a; Lian et al., 2022).

The effect of B doping on the kinetics of the deintercalation process of K ions on the electrode was further analysed using the AC impedance technique. The Nyquist curves and corresponding equivalent circuits for BZPC and ZPC after 10 cycles of charging and discharging at 10 mA g^−1^ were shown in Figure 6B. The compressed semicircle of the Nyquist curves in the mid-to-high frequency region corresponds to the charge-transfer resistance (*R*_*ct*_) and the ion transfer resistance through the SEI layer (*R*_*SEI*_), while the oblique line in the low-frequency region reflects the ion diffusion inside the active material (Wang et al., 2018a; Wu et al., 2019b). The parameters of the equivalent circuits were obtained by fitting the Nyquist curves. The diffusion coefficient of potassium ions (*D*_K_) was calculated using $D_{K^{+}}=0.5{\left(\frac{RT}{n^{2}F^{2}AC\sigma }\right)}^{2}$ , where *n* is the K ion transfer number, *R* and *F* are the Avogadro and Faraday constants respectively, *T* is the absolute temperature during the test, *A* is the reaction area of the electrode, *C* is the concentration of potassium ions in the electrode, and *σ* is the Warburg impedance coefficient (Gao et al., 2019). The fitting results indicated that B doping had a minor effect on the Ohmic resistance (*R*_*s*_) (Gao et al., 2019). The SEI film impedance decreased from 394 Ω (ZPC) to 290 Ω (BZPC), charge-transfer impedance dropped from 2,546 Ω (ZPC) to 1,629 Ω (BZPC), and potassium-ion diffusion coefficient increased from 3.57 × 10^−17^ cm^2^ s^−1^ (ZPC) to 3.61 × 10^−14^ cm^2^ s^−1^ (BZPC), indicating that B doping effectively improved the electrode kinetics of pine-cone carbon. It was speculated that B doping produced abundant active sites and defects to enhance the adsorption of K ions and expanded the interlayer distance to promote the intercalation and diffusion of K ions (Lian et al., 2022). In addition, the high specific surface area of B-doped pine-cone carbon increased the electrode/electrolyte contact area and reduced electrode polarisation, while its 3D hierarchical porous structure facilitated rapid penetration and migration of the electrolyte at high rates (Li et al., 2017; He et al., 2020; Tao et al., 2020).

## Conclusion

In this study, B-doped 3D hierarchical porous carbon (BZPC) was prepared using pine-cones as the raw material and zinc chloride and boric acid as the dopant sources. Electrochemical testing of BZPC as the anode of a KIB showed excellent cycling stability, high reversible capacity and excellent rate capability. The superior performance is attributed to the unique 3D hierarchical porous structure imparted by B doping, which generated numerous defects and active sites for adsorption of K ions, expanded the interlayer spacing, and mitigated the volume expansion caused by the intercalation of relatively large K ions into the carbon skeleton, thus maintaining structural integrity and providing an efficient electrolyte diffusion path. *Ex-situ* HRTEM and Raman spectroscopy measurements revealed reversible changes in interlayer spacing and degree of disorder during insertion/extraction of potassium ions. In addition, SEM characterisation indicated that the electrode material maintained structural stability under high current density and prolonged cycling. This research provides a new direction for designing biomass-derived carbon materials with excellent electrochemical properties, and provides a new strategy for manufacturing low-cost electrode materials for the energy storage industry with low environmental impact.

## Data Availability

The original contributions presented in the study are included in the article/Supplementary Material, further inquiries can be directed to the corresponding author.
