# Supplementary material for: Boron-Doped Pine-Cone Carbon With 3D Interconnected Porosity for Use as an Anode for Potassium-Ion Batteries With Long Life Cycle
Source: Front Chem. 2022 Jul 6;10:953782. doi: 10.3389/fchem.2022.953782 (PMC9296776; doi:10.3389/fchem.2022.953782)
Supplement: Supplementary file 1 [file DataSheet1.pdf]

Supplementary data

Boron-doped pine-cone carbon with 3D interconnected porosity for use as a anode for potassium-ion batteries with long life cycle

Jian-Fang Lu[a b], Ke-Chun Li[c], Xiao-Yan Lv[d], Hong-Xian Kuai[a], Jing Su[a], Yan-Xuan Wen[a e*],

[a]School of Chemistry and Chemical Engineering, Guangxi University, Nanning 530004, Guangxi, China.

[b]School of Chemistry and Chemical Engineering, Guangxi MINZU University, Nanning 530006, Guangxi, China.

[c]School of Materials and Environment, Guangxi MINZU University, Nanning 530006, Guangxi, China.

[d]The New Rural Development Research Institute, Guangxi University, Guangxi University, Nanning 530004, Guangxi, China.

[e]Guangxi Key Laboratory of Processing for Non-ferrous Metallic and Featured Materials, Guangxi University, Nanning 530004, Guangxi, China.

[*] Corresponding author. School of Chemistry and Chemical Engineering, Guangxi University, Nanning 530004, Guangxi, China.
E-mail address: wenyanxuan@vip.163.com (X.Y. Wen).

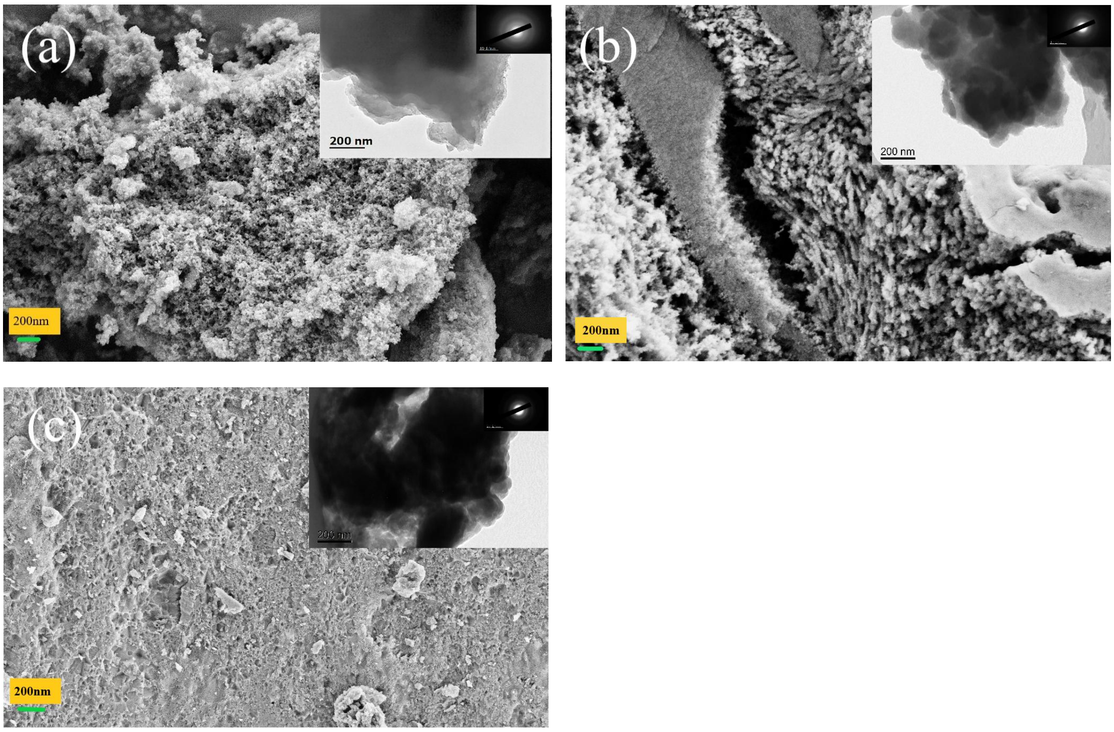

Figure S1. SEM and TEM images (inset) of the prepared samples:(a) ZPC, (b) BPC, and (c) PC.

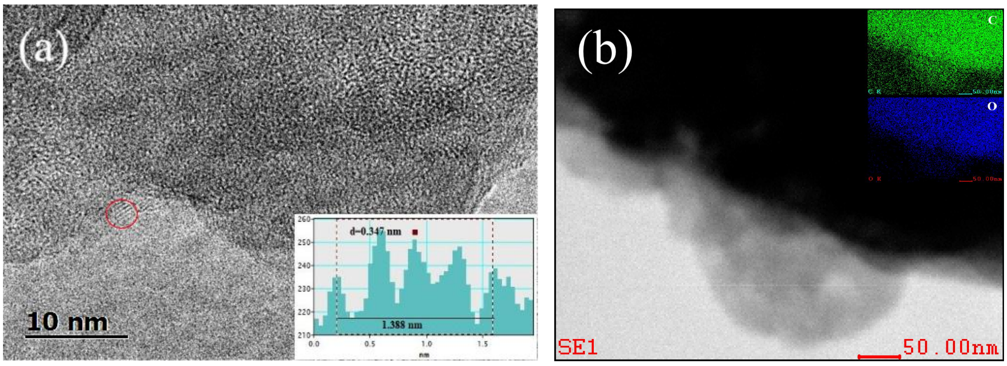

Figure S2. (a) HRTEM image . (b) EDS map of ZPC.

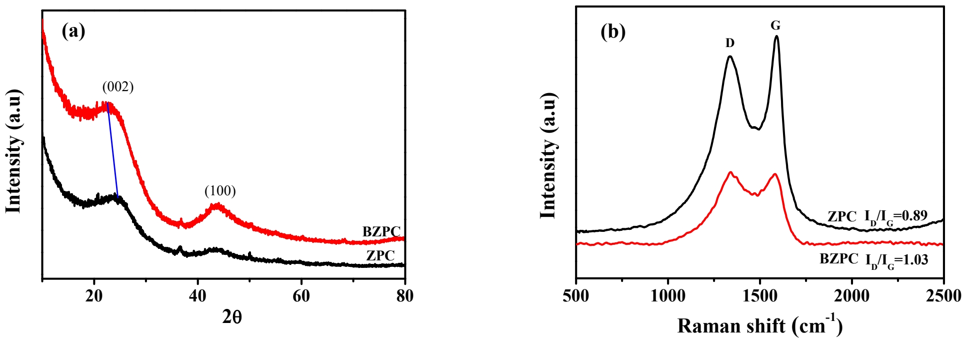

Figure S3. (a) XRD and (b) Raman spectra of the prepared ZPC and BZPC.

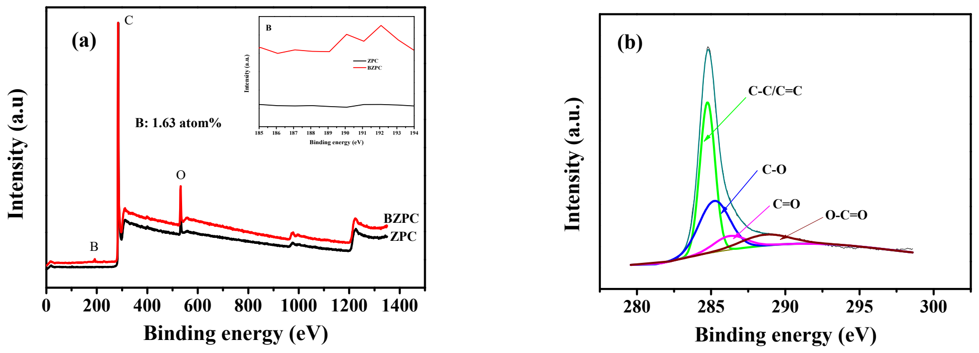

Figure S4. (a)XPS of the prepared ZPC and BZPC. (b) High-resolution C1s spectra of ZPC.

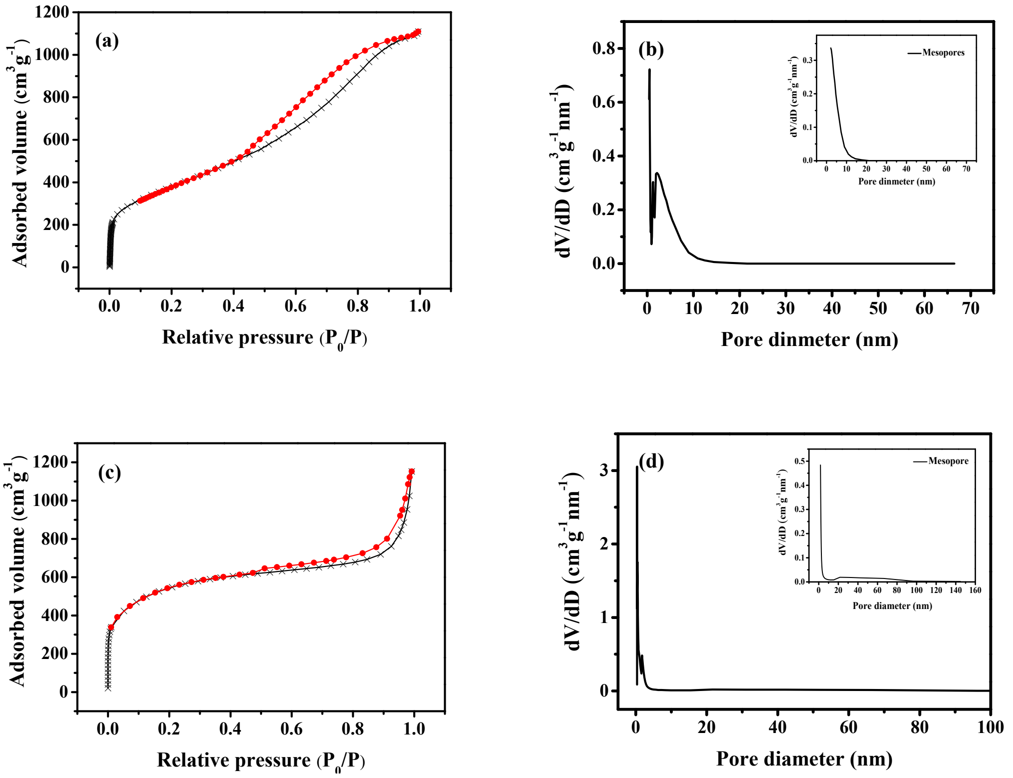

Figure S5. N_2 adsorption–desorption isotherms and pore-size distribution of (a, b) ZPC and (c, d) BZPC (inset of pore distribution calculation by BJH model).

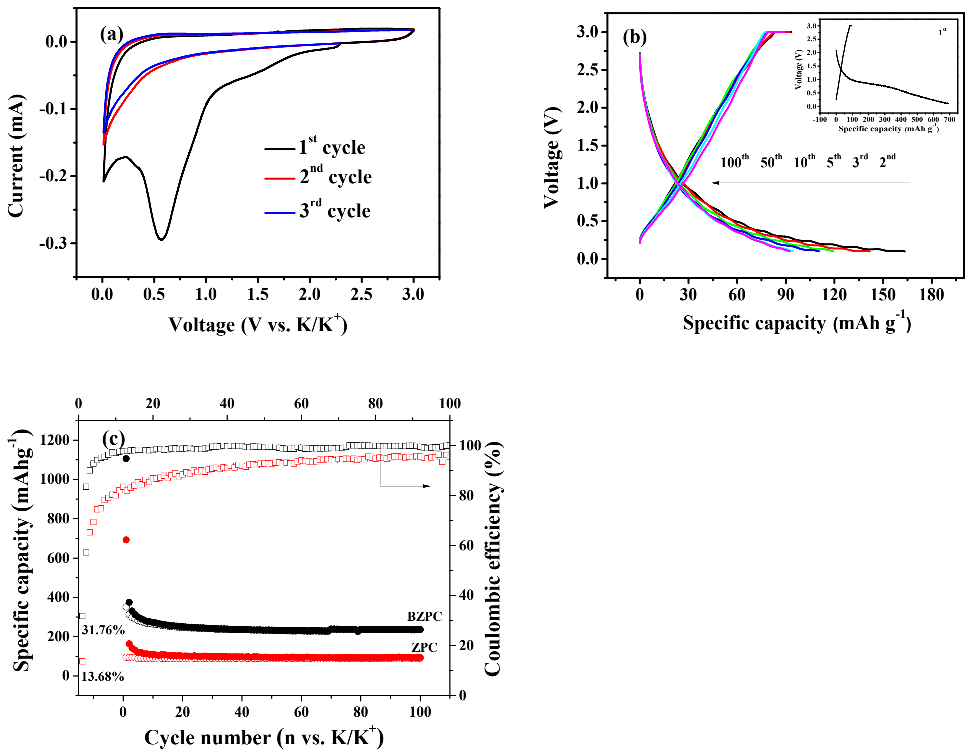

Figure S6. (a) CV curves of ZPC at 0.1 mV·s^{-1}. (b) galvanostatic charge/discharge profiles of different cycles of ZPC at 50 mA·g^{-1} (inset: 1st cycle). (c) cycle stability at 50 mA·g^{-1}.

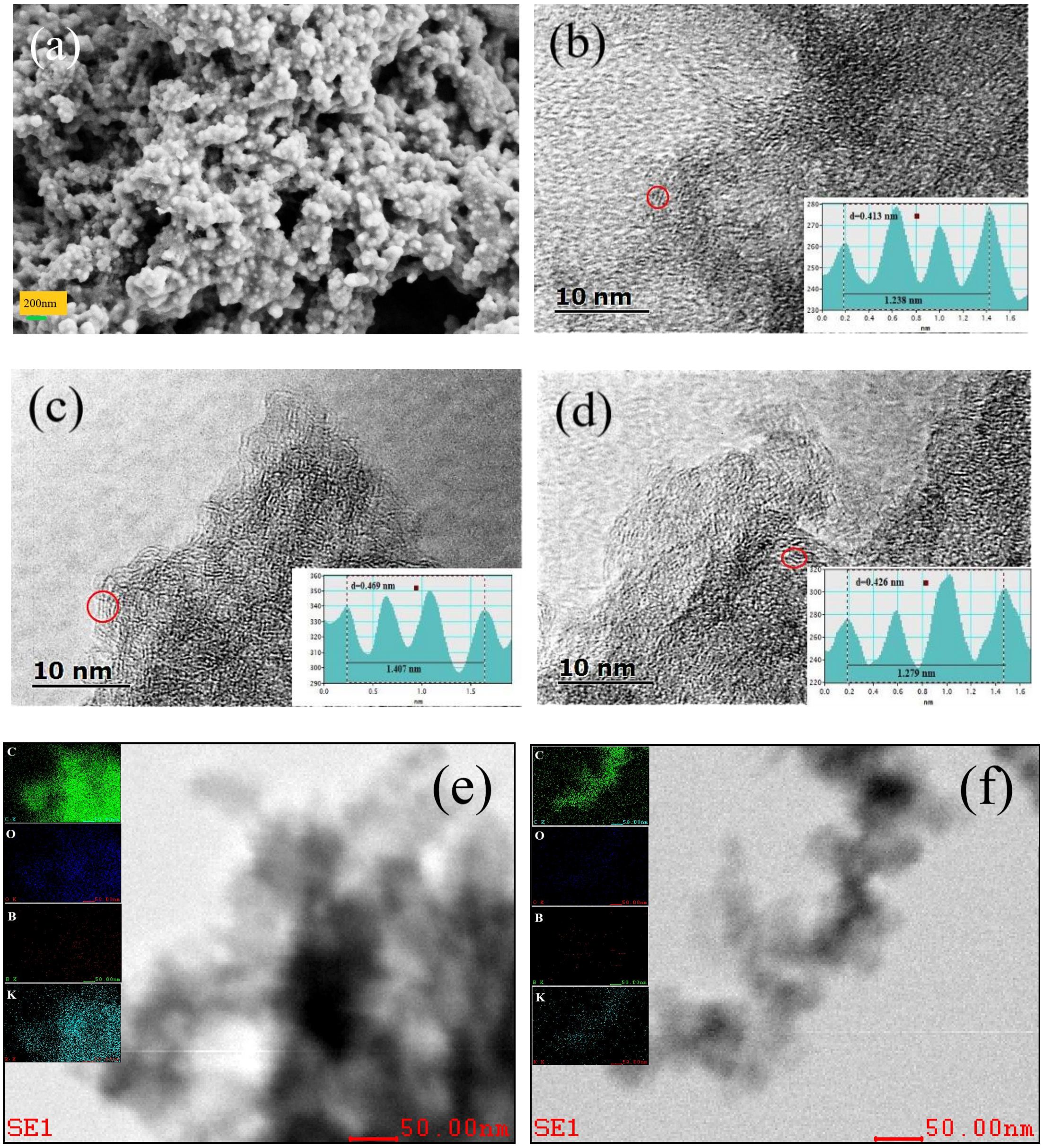

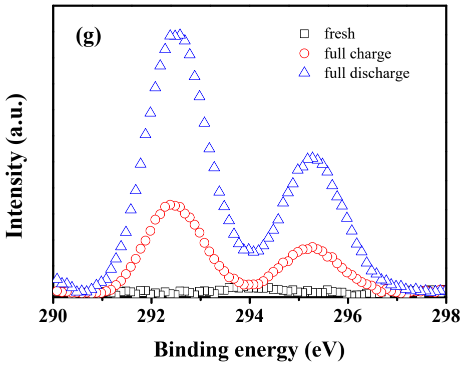

Figure S7. Effect of cycling on the anode microstructure. SEM images of (a) ZPC after 1000 cycles before testing. Ex-situ HRTEM of BZPC (b) before testing, at the (c) fully discharged state, and (d) fully charged state. EDS images of BZPC at the (e) full discharged state and (f) full charged state (10th cycle). Ex-situ XPS spectra of BZPC at various states.

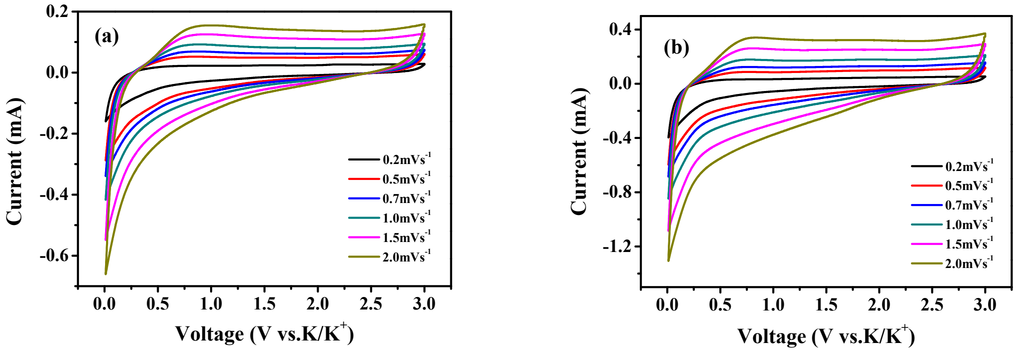

Figure S8. CV curves of (a) ZPC and (b) BZPC.
